# Transcriptional analysis of the mammalian heart with special reference to its endocrine function

**DOI:** 10.1186/1471-2164-10-254

**Published:** 2009-06-01

**Authors:** Monica Forero McGrath, Adolfo J de Bold

**Affiliations:** 1 Department of Cellular Molecular Medicine, Faculty of Medicine, Cardiovascular Endocrinology Laboratory, University of Ottawa Heart Institute, Ottawa, Canada; 2 Department of Pathology and Laboratory Medicine, Faculty of Medicine, University of Ottawa, Ottawa, Canada

## Abstract

**Background:**

Pharmacological and gene ablation studies have demonstrated the crucial role of the endocrine function of the heart as mediated by the polypeptide hormones ANF and BNP in the maintenance of cardiovascular homeostasis. The importance of these studies lies on the fact that hypertension and chronic congestive heart failure are clinical entities that may be regarded as states of relative deficiency of ANF and BNP. These hormones are produced by the atrial muscle cells (cardiocytes), which display a dual secretory/muscle phenotype. In contrast, ventricular cardiocytes display mainly a muscle phenotype. Comparatively little information is available regarding the genetic background for this important phenotypic difference with particular reference to the endocrine function of the heart. We postulated that comparison of gene expression profiles between atrial and ventricular muscles would help identify gene transcripts that underlie the phenotypic differences associated with the endocrine function of the heart.

**Results:**

Comparison of gene expression profiles in the rat heart revealed a total of 1415 differentially expressed genes between the atria and ventricles based on a 1.8 fold cut-off. The identification of numerous chamber specific transcripts, such as ANF for the atria and Irx4 for the ventricles among several others, support the soundness of the GeneChip data and demonstrates that the differences in gene expression profiles observed between the atrial and ventricular tissues were not spurious in nature. Pathway analysis revealed unique expression profiles in the atria for G protein signaling that included Gα_o1_, Gγ_2 _and Gγ_3_, AGS1, RGS2, and RGS6 and the related K^+ ^channels GIRK1 and GIRK4. Transcripts involved in vesicle trafficking, hormone secretion as well as mechanosensors (e.g. the potassium channel TREK-1) were identified in relationship to the synthesis, storage and secretion of hormones.

**Conclusion:**

The data developed in this investigation describes for the first time data on gene expression particularly centred on the secretory function of the heart. This provides for a rational approach in the investigation of determinants of the endocrine of the heart in health and disease.

## Background

In all mammals, atrial cardiac muscle cells (cardiocytes) display phenotypic differences with their ventricular counterparts owing to the endocrine function of the former. Atrial cardiocytes contain secretory-like granules known as *specific atrial granules*, which co-store two polypeptide hormones, atrial natriuretic factor (ANF or ANP, Nppa) and B-type natriuretic peptide (BNP, Nppb), referred to as cardiac natriuretic peptides (NP).

The increase in atrial NP gene expression and secretion observed during hemodynamic overload is viewed as a cardioprotective mechanism based on the capacity of ANF and BNP to reduce load by modulating renal function, vessel tone, as well as the renin-angiotensin-aldosterone and the sympathetic nervous systems, among others.

Considerable effort has been focused on the elucidation of the mechanistic underlying of ANF and BNP gene expression and secretion by the atrial cardiocytes. The majority of these investigations have concentrated on pharmacological interventions but much remains to be determined regarding specific genes involved in cardiocyte secretory function. This information is important however, to understand why under chronic conditions of hemodynamic overload such as in chronic congestive heart failure, the secretion of ANF and BNP is insufficient as demonstrated by the fact that patients that receive exogenous hormone benefit from the unloading of the heart induced by therapeutic administration of either of the hormones [1]. Through the present work we have defined genes that may be fitted within a more detailed view of cardiac endocrine function.

## Results

### Differentially expressed genes between atrial and ventricular tissues

**Figure 1:**
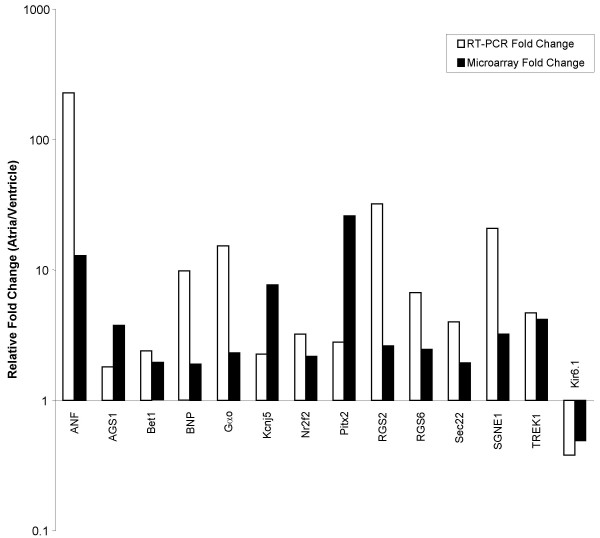
**Comparison of microarray and real-time PCR results**. Relative fold change of 14 candidate genes are shown. The results demonstrate agreement between the microarray and RT-PCR data, although the degree of fold change can vary.

From the 31042 probe sets found on the microarray, an average of 59% and 56% of probes had present calls in the atria and ventricles, respectively. Based on a 1.8 fold change ratio criterion, 1415 probe sets were statistically differentially expressed between the atria and ventricles. Of these, 859 probe sets showed higher expression levels in the atrial samples and 556 probe sets showed higher expression levels in the ventricles. The degree of change of the differentially expressed probes ranged between the 1.8 fold minimum set cut-off and 245 fold (see Additional file 1). Chamber specific genes were found differentially expressed in our dataset. Sarcolipin, an atrial-specific Ca^+2 ^binding protein was expressed 245.97 fold higher in the atria than the ventricles. Other atrial specific genes include dickkopf homolog 3 (DKK3) (3.32 fold), ANF (12.88 fold), and myosin light chain 2a (26.19 fold). In the ventricles, four-and-a-half LIM domain 2 (8.63 fold), myosin light chain polypeptide 3 (14.78 fold) and Iroquois homeobox protein 4 (Irx4) (18.44 fold) were identified. Differential expression of 14 candidate genes was confirmed by real time-PCR. The results show excellent agreement between the microarray and PCR data (Figure 1). Several genes exhibited a higher fold change ratio when analyzed by PCR as compared to microarray.

### Functional classification

**Figure 2:**
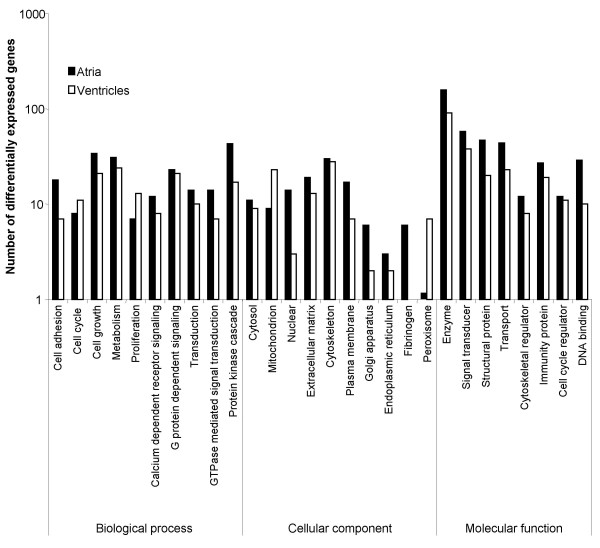
**Functional classification of differentially expressed genes found between the atria and the ventricles with known Gene Ontology annotations**. Note that the number of differentially expressed genes are plotted according to muscle type, i.e. atrium or ventricle.

Differentially expressed genes between the atria and the ventricles were functionally classified into four major Gene Ontology categories: biological process, cellular component, molecular function and unclassified. An individual transcript was included in several categories or subcategories because of its relevance to more than one category or subcategory. Out of the 1415 significantly differentially expressed probes, 837 were grouped as unclassified, i.e. had unknown annotations at the time of the study. Figure 2 illustrates the extent in gene expression differences found between the two heart tissues across functional classes.

#### Biological processes

**Figure 3:**
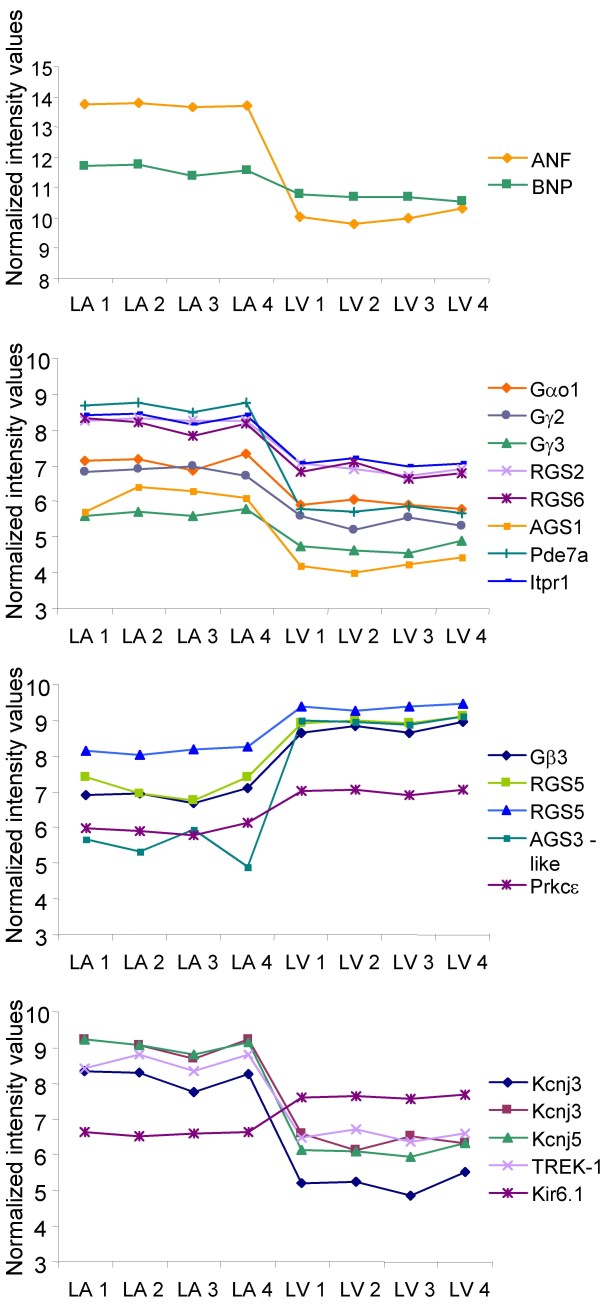
**Normalized intensity values of specific transcripts in all atrial and ventricular microarray replicates**. The data is displayed in four different panels for visual simplicity. Note that atrial and ventricular microarrays are labelled as LA(n) and LV(n), and the scale of the ordinate axis is different for the upper panel due to high signal intensity values for ANF and BNP. Also note that RGS5 and Kcnj3 are represented by two distinct probe sets.

Differentially expressed genes were similarly abundant in cell communication and signal transduction processes and they were mainly found involved in cell growth, metabolism, transport, G protein-dependent signaling, and protein kinase cascade (Figure 2). Differentially expressed G protein-dependent signaling transcripts include those grouped in subcategories such as glutamate signaling, G protein signaling, neuropeptide signaling pathway, and serotonin receptor signaling (Table 1). Regulators of G-protein signaling (RGS) RGS2 and RGS6 were more abundant in the atria (2.61 and 2.47 fold, respectively) while RGS5 was prevalent in the ventricles (2.33 fold) (see Figure 3). Other accessory proteins for G proteins that were found differentially expressed include periplakin (4.23 fold), Rasd1 (also known as Activator of G protein Signaling 1, AGS1) (3.75 fold) and β-site APP cleaving enzyme 2 (3.54 fold). These genes were more abundant in the atria while Rasd2 (3.32 fold) and AGS3-like (11.43) were more abundant in the ventricles. All these accessory proteins, including the RGS proteins interact with Gα_i/o _[2]. Small GTPase-mediated signal transduction transcripts found differentially expressed include regulators of membrane trafficking including Rab15 (24.53 fold), Rab34 (1.90 fold) and Rab40b (3.27 fold). The Rab escort protein choroidermia, known to interact with Rab3a and Rab27a, as well as the Rab27a effector protein synaptotagmin-like 2 were abundantly expressed in the atria by 1.86 and 32.25 fold, respectively. The latter is known to bind and regulate the GTP-bound form of Ras-related protein Rab27a, which has been shown to positively regulate the exocytosis of secretory granules in pancreatic beta cells and pituitary tissue [3].

**Table 1:** List of differentially expressed genes classified into G protein dependent signaling and related functional groups

**Functional Group/Probe set ID**	**Gene Description**	**Fold change**
**G Protein signaling**		
1369740_at	Potassium inwardly-rectifying channel, subfamily J, member 3	7.76
1368560_at	Potassium inwardly-rectifying channel, subfamily J, member 5	7.68
1369741_at	Potassium inwardly-rectifying channel, subfamily J, member 3	6.35
1369273_a_at	Natriuretic peptide receptor 3	6.02
1396249_at	Natriuretic peptide receptor 3	4.06
1387803_at	Protein phosphatase 2, regulatory subunit B, beta isoform	3.94
1367920_at	Endothelial differentiation, sphingolipid G-protein-coupled receptor, 5	3.27
1367791_at	Receptor (calcitonin) activity modifying protein 1	3.05
1392905_at	Guanine nucleotide binding protein, gamma 2	2.74
1368879_a_at	Guanine nucleotide binding protein, alpha o	2.31
1369377_at	Hypocretin receptor 2	2.00
1373202_at	Guanine nucleotide binding protein, gamma 3	1.96
1369647_at	Calcitonin receptor-like	-1.82
1369115_at	Adrenergic receptor, beta 2	-1.83
1368660_at	cAMP-regulated guanine nucleotide exchange factor I	-1.93
1388109_at	G protein-coupled hepta-helical receptor Ig-Hepta	-1.95
1370449_at	G protein-coupled receptor 105	-2.20
1368534_at	Adrenergic receptor, alpha 1d	-2.34
1398530_at	guanine nucleotide binding protein (G protein), gamma 11	-2.34
1368300_at	Adenosine A2a receptor	-2.46
1370522_at	Glucagon receptor	-3.07
1395473_at	Guanine nucleotide binding protein, beta 3	-3.65
1387671_at	Secretin receptor	-4.31
		
**Accessory proteins for G proteins**		
1391187_at	Periplakin (predicted)	4.23
1387908_at	RAS, dexamethasone-induced 1 (Rasd1/AGS1)	3.75
1377390_at	Beta-site APP-cleaving enzyme 2	3.54
1387074_at	Regulator of G-protein signaling 2	2.61
1379517_at	Regulator of G-protein signaling 6	2.47
1369957_at	Regulator of G-protein signaling 5	-2.33
1370372_at	RASD family, member 2	-3.32
1372383_at	G-protein signaling modulator 1 (AGS3-like, C. elegans)	-11.43
		
**Neuropeptide signaling pathway**		
1367992_at	Secretory granule neuroendocrine protein 1	3.20
1382967_at	G protein-coupled receptor 64	2.50
1369377_at	Hypocretin receptor 2	2.00
1368428_at	X-prolyl aminopeptidase (aminopeptidase P) 2, membrane-bound	-1.99
1371696_at	G protein-coupled receptor 56	-2.16
1367949_at	Preproenkephalin	-13.55
		
**Serotonin receptor signaling**		
1369125_at	5-hydroxytryptamine (serotonin) receptor 2A	7.84
1369124_at	5-hydroxytryptamine (serotonin) receptor 2A	1.88

Positive fold change values indicate a higher abundance in the atria as compared to the ventricles, while negative fold change values demonstrate a higher ventricular abundance in comparison to the atria.

Genes involved in secretion included signal sequence receptor gamma (SSR3) (1.82 fold), blocked early in transport 1 homolog (S. cerevisiae) (Bet1) (1.93 fold), vesicle trafficking protein like 1 (Sec22) (1.93 fold), synapsin III (1.93 fold) and annexin A1 (2.04 fold). These genes were found more abundant in the atria when compared to the ventricles.

#### Cellular component

Within the Gene Ontology category of cellular components, the intracellular components subcategory contained the largest amount of differentially expressed genes (n = 156) in comparison to the extracellular (n = 37) and the membrane components (n = 37). Cellular components that had prominent atrial abundant transcripts included those associated with the plasma membrane, the endoplasmic reticulum (ER), the Golgi apparatus, and the nucleus, while the ventricles were associated with abundant transcripts found within the mitochondrion and peroxisome components (Figure 2). ER and Golgi-associated genes that were abundantly expressed in the atria include stearoyl-CoA desaturase 1 (20.81 fold) and 2 (2.21 fold), inositol 1,4,5-triphosphate receptor 1 (2.44 fold), ribosome associated membrane protein 4 (2.22 fold), Sec11 like 3 (2.05 fold), as well as SSR3. Transcripts involved in ER to Golgi vesicle-mediated transport include the previously mentioned genes Sec22 and Bet1. Other atrial abundant Golgi resident proteins include islet cell autoantigen of 1 (Ica1) (4.63 fold), transforming growth factor-β2 (TGFβ2) (3.89 fold), nucleobindin 2 (2.35 fold) and Golgi transport 1 homolog B (Golt1b) (2.60 fold).

#### Molecular function

**Figure 4:**
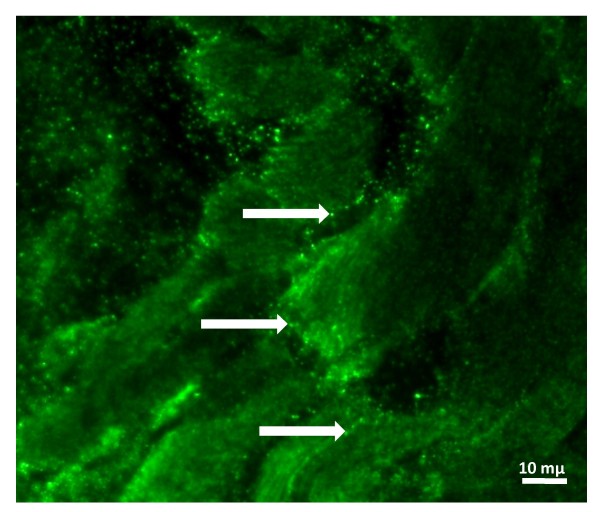
**Paraffin-embedded, paraformaldehyde-fixed atrial tissue section stained with SGNE1-FITC antibody showing punctuated fluorescence structures suggesting adrenergic varicosities (white arrows)**.

The molecular function subcategories that were most notably differentially expressed between atrial and ventricular muscles included enzyme, signal transducer, structural protein, and transport (see Figure 2). The signal transducer subcategory is composed of ligands and receptors (see Additional file 2 and Additional file 3 for complete list of genes). The cytokines, frizzled receptor ligands, growth factor receptor ligands as well as hormone receptor ligands subcategories were also significantly differentiated. Differentially expressed genes involved in hormone, hormonal activity, and hormone receptor ligand subcategories, including ANF (12.88 fold) and BNP (1.89 fold), as well as secretory granule neuroendocrine protein 1 (SGNE1) (3.20 fold) are listed in Table 2. Preliminary immunohistochemistry analysis localized SGNE1 in structures compatible with adrenergic varicosities (Figure 4). Differentially expressed transcription factors/DNA binding proteins were more abundant in the atria than in the ventricles (see Additional file 4 for complete list).

**Table 2:** List of differentially expressed genes possessing hormone, hormonal activity, or hormone receptor ligand Gene Ontology annotations

**Probe Set ID**	**Gene Description**	**Fold change**
1367564_at	Natriuretic peptide precursor type A (ANF)	12.88
1367616_at	Natriuretic peptide precursor type B (BNP)	1.89
1371400_at	Thyroid hormone responsive protein	38.59
1387396_at	Hepcidin antimicrobial peptide	11.24
1369484_at	WNT1 inducible signaling pathway protein 2	4.79
1387625_at	Insulin-like growth factor binding protein 6	3.61
1368883_at	Top of Form Nephroblastoma overexpressed gene Bottom of Form	3.51
1367992_at	Secretory granule neuroendocrine protein 1 (SGNE1)	3.20
1368367_at	CUB and zona pellucida-like domains 1	3,17
1368367_at	Integral membrane-associated protein 1	3.16
1376734_at	Nephroblastoma overexpressed gene	2.79
1372168_s_at	Insulin-like growth factor binding protein 6	2.25
1367631_at	Connective tissue growth factor	2.20
1389651_at	Apelin, AGTRL1 ligand	2.00
1387219_at	Adrenomedullin	1.87
1367894_at	Growth response protein (CL-6)	1.82
1370563_at	3-alpha-hydroxysteroid dehydrogenase	-1.92
1368428_at	X-prolyl aminopeptidase (aminopeptidase P) 2, membrane-bound	-1.99
1396101_at	Stanniocalcin 1	-2.14
1368468_at	Cytochrome P450, subfamily 11A	-3.97
1367949_at	Preproenkephalin, related sequence	-13.55

Positive fold change values indicate a higher abundance in the atria as compared to the ventricles, while negative values demonstrate a higher ventricular abundance in comparison to the atria.

In general, genes coding for transport proteins were more abundant in the atria than in the ventricles. Potassium channels, which were most abundant in the atria included potassium channel subfamily K member 2 (Kcnk2 or TREK-1), as well as potassium inwardly-rectifying channel, subfamily J, member 3 (Kcnj3) and 5 (Kcnj5) (Table 3). The ventricles were most abundant in the potassium inwardly-rectifying channel, subfamily J, member 8 (Kcnj8 also known as Kir6.1) channel subunit.

**Table 3:** List of differentially expressed genes encoding for potassium channels

**Probe Set ID**	**Gene Description**	**Symbols**	**Fold Change**
1369740_at	Potassium inwardly-rectifying channel, subfamily J, member 3	Kcnj3 or GIRK1	7.78
1368560_at	Potassium inwardly-rectifying channel, subfamily J, member 5	Kcnj5 or GIRK4	7.67
1369741_at	Potassium inwardly-rectifying channel, subfamily J, member 3	Kcnj3 or GIRK1	6.36
1370342_at	Potassium channel, subfamily K, member 2	Kcnk2 or TREK-1	4.17
1368911_at	Potassium inwardly-rectifying channel, subfamily J, member 8	Kcnj8 or Kir6.1	-2.03

Positive fold change values indicate a higher abundance in the atria as compared to the ventricles, while negative values demonstrate a higher ventricular abundance in comparison to the atria.

### Pathway analysis

**Figure 5:**
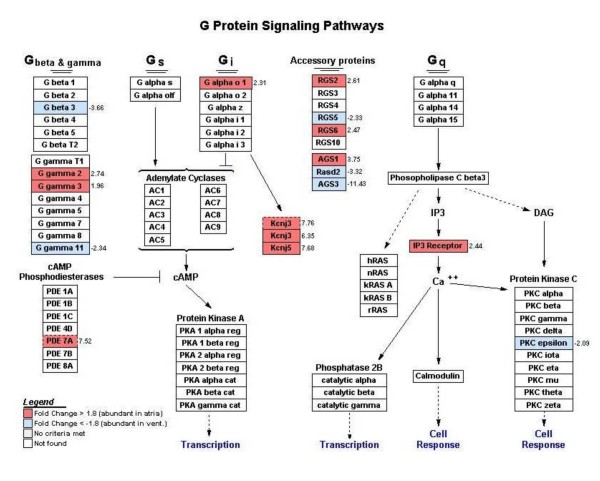
**G protein signaling pathways generated by GenMapp**. Genes labeled in red indicate a higher expression level in the atria as compared to the ventricles based on a 1.8 fold threshold cut-off. Genes labeled in blue indicate a higher expression level in the ventricles as compared to the atria based on a -1.8 fold threshold cut-off. Genes labeled in white were not found in the list of statistically significant differentially expressed genes that was uploaded in GenMapp.

Pathway analysis using the Gene Map Annotator and Pathway Profiler (GenMapp) software identified clusters of differentially expressed genes found within the same biological pathway. Visual representation of specific known pathways, referred as Mapps, revealed several Mapps in which atrial gene expression was predominant. These Mapps included pathways of fatty acid synthesis, prostaglandin synthesis, as well as eicosanoid synthesis, which shows a higher abundance of phospholipase A2 (PLA2) (3.28 fold), COX1 (2.11 fold) and COX2 (1.96 fold). In contrast, mitochondrial long-chain fatty acid β-oxidation and glucocorticoid metabolism pathways were dominant in ventricular samples. Mapps other than those from metabolic processes, revealed atrial regional specialization in regards to the classical component activation pathway and G-protein signaling pathway (Figure 5). The latter demonstrated a higher abundance of the Gα_o1 _subunit (Gnao), Kcnj3 and Kcnj5 in the atrium compared to the ventricle by 2.3, 6.35 (second probe set = 7.76 fold) and 7.68 fold, respectively. Also, Gγ_2 _(Gng2), Gγ_3 _(Gng3) (2.74 and 1.96 fold, respectively) and phosphodiesterase 7a (Pde7a) (7.52 fold) as well as inositol 1,4,5-triphosphate receptor 1 (Itpr1) (2.44 fold) were highly expressed in the atria while protein kinase C epsilon (Prkcε) was more abundant in the ventricles by 2.09 fold. Gβ subunits 1, 2, and 5 were present in both atria and ventricles at roughly similar levels while Gβ_3 _(Gnb3) was expressed at higher levels in the ventricles (3.66 fold).

## Discussion

Mammalian atrial and ventricular cardiocytes display striking differences in phenotype. The bulk of atrial cardiocytes have, in addition to features commonly associated with striated cardiac muscle cell cytology, organelles that are normally found in cells engaged in the synthesis, vectorial transport and secretion of polypeptide hormones. These include an abundance of rough endoplasmic reticulum, a highly developed Golgi complex and storage granules containing hormonal products. These cytological features led to a series of past investigations that culminated in the discovery of the endocrine function of the heart [4], which is largely mediated by the polypeptide hormones ANF and BNP.

From the data presented here it is evident that the phenotypic differences between the atria and the ventricles results from underlying differences in degree or presence/absence of several specific genes. Despite this complex background of differential expression, the microarray data reproducibility observed in different biological replicates was excellent.

The proportion of probes that were called present, as well as the number of differentially expressed genes was higher in the atria than in the ventricles. This observation could be related to a higher functional complexity of the atrial tissue, which include contraction, excitation, conduction, secretion and the presence of a profuse innervation [5,6]. The detection of numerous known chamber specific transcripts [6-9] supports the robustness of the GeneChip data and demonstrates that the differences in gene expression profiles observed between the atrial and ventricular tissues are not spurious in nature. A comparative analysis of the differentially expressed genes identified in other studies that are in common with our data is presented in the Additional file 5. Several genes identified in these studies by others are in agreement with our data, even though there are experimental differences (such as species, gender and tissue selection) as well as data analysis between studies. However, none of these previous studies focused specifically on gene expression profiles relating to the endocrine function of the heart.

Based on atrial and ventricular phenotypical differences, the strategy used in the data mining process was to identify genes that had Gene Ontology annotations relating to hormone secretion, hormone activity, vesicle/granule packaging and transport. Screening for Gene Ontology terms classified as Golgi apparatus and ER cellular components was also undertaken in an effort to identify genes involved in the regulation of NP packaging and secretion. Differentially expressed transcription factors as well as transcripts coding for proteins involved in signal transduction such as ligands and receptors were also of great interest. Finally, hormone secretion can be modulated/regulated by ion channels and mechanosensors, hence genes coding for transport proteins were also of interest. Differentially expressed genes coding for proteins involved in metabolic processes as well as contractile proteins were not specifically looked at since these differences are expected to be found between the atrial and ventricular tissues but are not thought to be involved in the endocrine function of the heart. In general, functional classification of the differentially expressed genes according to their cellular components, identified in our study, parallels the results obtained by Barth et al.,[5], which include ventricular abundance of mitochondrial genes as well as the identification of numerous atrial abundant ER and Golgi apparatus transcripts, such as Sec22 and Rab34. The larger number of probes hybridizing to cellular components involved in secretory function agrees with the phenotypic and functional differences between atrial and ventricular muscle. For example, atrial cardiocytes were more abundant in the transcripts coding for ANF and BNP than the ventricles. Correspondingly, ANF transcript abundance, which constitutes one to three percent of total atrial mRNA [10] was found to provide for the top one percent of the GeneChip^® ^signal intensities. Saturation of the fluorescent signal is thought to have occurred for ANF probe sets, which explains the plateau level of 12 fold difference between atrial vs. ventricular tissues as compared to 163 fold with RT-PCR (Figure 1). Oligonucleotide probe saturation can occur when the labeled cRNA is at high enough levels to result in an almost 100% probe/cRNA hybridization complex. Any other increase in expression would not be measurable due to the olignucleotide hybridization saturation. It is difficult to estimate the amount of cRNA needed to saturate the oligonulceotide probes since the specific number of oligonucleotide probes tiled onto the 11^2 ^micron surface area of the GeneChip^® ^is proprietary information.

Storage granule-associated proteins identified in this study include Ica1 [11] and SGNE1. The evolutionary conserved gene Ica1, was shown to be abundant in human pancreas and heart, and moderately expressed in brain [12]. Variable expression of 5'-untranslated region exons from the Ica1 gene was shown to be tissue specific, where cardiac tissue distinctively expressed the exon B2 transcript variant [13]. The original observation that Ica1 expression levels were high in the heart but null in skeletal muscle lead Pietropaolo et al., [12] to postulate that Ica1 was present in selective cells. We demonstrate here that Ica1 is expressed specifically in the atria given that all four ventricular replicates exhibited an absent detection call for this transcript (an absent call indicates that the microarray expression level for a particular transcript was below the threshold of detection).

Several studies have been able to co-localize SGNE1, a member of the secretogranin family, with neuropeptides and hormones in various tissues that are primarily composed of neuronal or endocrine cells, as well as tissues known to contain a sub-population of neuroendocrine cells [14]. RT-PCR analysis further confirmed atrial abundance of SGNE1 (Figure 1). SGNE1 has not been previously associated with the atria or the heart. Our own immunofluorescence preliminary data suggest that SGNE1 protein is localized in structures compatible with adrenergic varicosities (Figure 4).

Differentially expressed transcription factors/DNA binding proteins that were more abundant in the atria than in the ventricles included numerous orphan nuclear receptors. Two out of the three Affymetrix probe sets assaying for nuclear receptor subfamily 2, group F, member 2 (Nr2f2) transcripts were identified in the atrial abundant gene list. Nr2f2 expression pattern is in agreement with previously published data (Additional file 5) and real-time PCR analysis further validated its atrial abundance (Figure 1). Other atrial abundant transcription factors include paired-like homeodomain transcription factor 2 (Pitx2), which is involved in atrial septation; T-box protein 5 (Tbx5), a known activator of the ANF promoter; and early growth response 1 (Egr1), which is up-regulated during endothelin, angiotensin II, ischemia and stretch treatments. Hairy/enhancer-of-split related with YRPW motif 1 (Hey1), as well as its activator Notch1, were more abundant in the ventricles than in the atria. Hey1 is thought to repress ANF expression in the ventricles [15]. The known tissue specificity of several of these transcription factors further illustrates the soundness of the dataset and the identification of novel atrial abundant DNA binding proteins warrants further attention.

Of special importance to the understanding of the regulation of hormone secretion by the atria are genes that encode for molecular species involved in mechanical aspect of atrial activity given that a major stimulus for ANF and BNP secretion is atrial muscle stretch. This phenomenon is referred to as "stretch-secretion coupling" [16]. Questions still remain regarding the exact stimulus perceived by the atrial cardiocytes as well as the precise signal transduction cascade that leads to an increase in ANF and BNP secretion following stretch. Therefore, the finding that mechanoreceptors (mechanically gated ion channels) as well as mechanosensors (PLA2) are most abundant in the atria is central to the generation and the testing of hypothesis. Because some prostaglandins are known to be potent stimulators of ANF synthesis and secretion [17], it is of interest that the mechanosensor PLA2 is abundantly expressed in the atria. Activation of PLA2 following plasma membrane stretch could cause the release of arachidonic acid, a substrate for prostaglandin synthase.

The rapid secretory response following stretch of atrial cardiocytes as compared to the response to other ANF secretion agonists such as endothelin-1 (ET-1) [18] has long suggested that the phenomenon of stretch-secretion coupling is related to mechanosensitive ion channels. It has been suggested [19-22] that K_ATP _channels are involved in stretch-secretion coupling. These channels are stretch-sensitive [23], are known to couple to G proteins, including Gα_o _[24,25], and have been associated with other secretory processes [26-28]. The results and conclusions reached from investigations on the involvement of K_ATP _channels on ANF secretion have often been contradictory. An inspection of the literature suggests that a likely reason for these discrepancies may lie on differences in experimental systems (in vivo or in vitro, perfused or non perfused tissues, etc.) [19-22]. Most importantly, pharmacological studies have been made with little reference to the several candidate ion channels that may be present in the atria. The present investigation shows that Kir6.1 is significantly expressed in the ventricles. The ATP binding cassette SUR2, whose expression level (1.79 fold) didn't meet the fold cut-off criteria was more abundant in the ventricles, and, while not significant, Kir6.2 and SUR1 had marginally higher expression in atria.

Three other channels had significantly higher levels of expression in the atria over the ventricles. These included the inward rectifiers Kcnj3, Kcnj5 and the background channel TREK-1. Kcnj3,5 channels, which are activated by G protein coupled receptors (GPCRs) coupled to pertussis toxin (PTX) sensitive G_i/o _proteins, exhibit mechanosensitive properties and might participate in a mechanoelectrical feedback pathway regulating ANF and BNP secretion [29,30]. This view is in line with our recent finding that PTX can abolish stretch secretion coupling [31]. The background channel TREK-1 produces an outwardly rectifying current and is a member of the 2 pore domain potassium (K_2P_) channel family. TREK-1 has been characterized as a stretch-activated membrane channel. It is primarily expressed in the central nervous system but has been found in cardiac tissues [32] as well as in specific endocrine cells, such as adrenocortical cells [33]. Atrial abundance of TREK-1 was further validated by RT-PCR (Figure 1). Immunohistochemical analysis localized TREK-1 protein in isolated adult rat atrial cardiocytes and it is suggested that TREK-1 may act as a regulator of ANF secretion [34].

The complex signal transduction pathways resulting from the activation of GPCRs is in part orchestrated by an assorted population of G proteins, assembled from a various combinations of 20 Gα, 5 Gβ and 12 Gγ isoforms [2]. Pathway analysis of the microarray data revealed differential expression of genes involved in G protein signaling pathways between atrial and ventricular tissues. The G protein Gα_o _isoform 1 was found more abundant in the atria by both microarray and RT-PCR analyses and was demonstrated by immunohistochemistry to co-localize with ANF in atrial granules and in the sarcolemma [31]. Differential expression of the Gβ and Gγ subunit isoforms was also observed between the atria and ventricles but the functional significance of the differential G protein subunit diversity is not well known.

The identification of accessory proteins involved in the modulation of G protein signal transduction has helped to gain insight into the mechanisms involved in the specificity of a cellular response from an assortment of external stimuli. RGS proteins are capable of inhibiting signaling by acting as a GTPase Accelerating/Activating Protein (GAP), which accelerate the intrinsic rate of GTP hydrolysis of the G_αi/o _subunit [35]. The preponderance of genes encoding for RGS proteins appears to be tissue specific. RGS2 and RGS6 were abundantly expressed in the atria while RGS5 was found at higher levels in the ventricles. These findings are confirmed by other studies in which RGS mRNA and protein levels were measured within atrial and ventricular cardiocytes [35-37].

In addition to the previously mentioned accessory proteins, in this work we identified the atrial specific transcript Rasd1, also known as AGS1. AGS1 was found more abundant in the atria by both microarray and RT-PCR analyses. In the absence of receptor stimulation, AGS1, a member of the Ras subgroup of small G proteins, specifically activates G_i/o _heterotrimeric signaling pathways by promoting nucleotide exchange on Gα_i/o _proteins and enhancing Gβγ uncoupling from Gα_i/o _[38]. Since stretch secretion coupling of ANF and BNP is mediated by PTX-sensitive Gα_i/o_, the differential expression of AGS1, as well as the other accessory proteins for G proteins identified in this investigation, is of particular interest. Due to AGS1 opposing effects on G_i/o _mediated signaling following agonist dependent or independent GPCR activation [39-42], AGS1 may help orchestrate the complex diversity and specificity of G protein signaling under different conditions.

## Conclusion

The data developed in this investigation describes for the first time data on gene expression particularly centred on the secretory function of the heart. Based on our data mining strategy, we narrowed down the list of the 1415 differentially expressed genes to a group of 115 genes. Additional data filtering generated a subset of 20 candidate genes, described herein, that are likely involved in the endocrine function of the heart. Of special interest is the relationship between G proteins of the inhibitory type, as well as their accessory proteins, and members of the inward rectifiers and the two pore domain K^+ ^channel superfamily. This investigation provides the basis of hypotheses formulation to further explore the endocrine function of the heart in physiological and pathophysiological conditions. Several lines of investigation are being pursued in our laboratory based on the findings described herein.

## Methods

### Total RNA extraction

The left atria appendage and the left ventricular free wall were obtained from thirteen normal male Sprague Dawley rats (225–275 g; Charles River Laboratories, Wilmington, MA), sacrificed by decapitation. The experimental protocol was carried out according to the Canadian Council on Animal Care Guide to the Care and use of Experimental Animals following approval by the University of Ottawa Animal Care Committee. The tissues were snap-frozen in liquid nitrogen and stored at -80°C. Atrial and ventricular tissues were grouped and then divided into 4 pools from which total RNA was extracted using Trizol^® ^(Invitrogen, Carlsbad, CA) following the manufacturer's instructions.

### Microarray hybridization

The quality of the extracted RNA from four independent pools of atrial and ventricular tissue was assessed using an Agilent Bioanalyzer (Agilent Technologies, Palo Alto, CA). Ten μg of total RNA was used to generate the first strand cDNA using the one cycle target labeling protocol. The biotin-labeled cRNA was prepared according to the manufacturer's protocol and hybridized to the GeneChip^® ^Rat Genome 230 2.0 Array (Affymetrix, Santa Clara, CA) which contains 31042 probe sets and analyses over 30000 transcripts from 28000 genes. Detailed description of the hybridization protocol is available at:

. Four biological replicates for each muscle type were generated. These steps were carried out by the Genome Québec Innovation Centre (Montreal, QC).

### Data analysis

The files that contain cell intensities (.CEL) were visually inspected with the dChip  software as a quality control measure in order to identify local image contamination prior to normalization. Preliminary expression summaries produced by Microarray Suite 5.0 (Affymetrix, Santa Clara, CA) (MAS5) software were used to generate scatterplots, to assess microarray reproducibility, and detection calls. The normalized signal intensities from the normal atrial replicates as well as from the normal ventricular replicates were visualized by pairwise scatterplots in which individual chips within the same group were plotted against each other (within-class). The majority of data points representing signal intensities for both atrial and ventricular tissues were highly correlated for the four replicates.

Further analysis was conducted using the expression level summary files computed by Robust Multiple-Array Averaging (RMA) which is implemented in Bioconductor . We used the RMA summary method, which generates background adjustments, quantile normalizations and summarizations of the raw scanned data in order to produce a measure of mRNA expression levels. It has been shown that RMA has superior precision, better estimation of fold change and provides higher specificity and sensitivity when analyzing fold changes to detect differentially expressed genes [43,44]. Differential expression of genes was considered to be significant based Significance Analysis of Microarrays (SAM)  with a False Discovery Rate (FDR) of 0.01%. Genes were considered biologically differentially expressed based on an up- or down-regulation of expression levels by at least 1.8 fold (log base 2 = 0.848). The complete filtered raw data can be found in Additional file 1. Sample hierarchical clustering was performed with TIGR MeV software  using the Pearson uncentered correlation with complete linkage. Pathway analysis, which identifies biologically relevant networks that exist among differentially expressed genes, was done by GenMAPP software . Functional classification was carried out using GeneSpring 7.0  and Netaffx  software. The microarray data has been deposited in the National Center for Biotechnology Information's Gene Expression Omnibus (GEO) and complies with MIAME standards (accession number: GSE5266).

### Real-time PCR

Real-time PCR was used to validate expression profiles of transcripts using the Roche LightCycler 480 relative quantification software. Standard curves were performed in triplicate for each primer pair. G6PD was used as the reference gene. RT-PCR was performed in quadruplet for each gene and the median crossing point was used to determine the concentration ratio, based on the relative standard curves for each target and reference gene. Most primer nucleotide sequences were obtained from the literature and validated by PRIMER3 . Primer sequences are listed in Table 4.

**Table 4:** Oligonucleotide sequences used for real-time PCR

**Name**	**Forward Primer**	**Reverse Primer**
ANF	GCCGGTAGAAGATGAGGTCA	GGGCTCCAATCCTGTCAATC
AGS1	GCGGCGAAGTCTACCAGTTG	TGTCTAAGCTGAACACCAGAATGA
Bet1	AAGCAAGTGGGAGAGCAGAA	GAGGAAAGAAACGGCCCTAC
BNP	TCTGCTCCTGCTTTTCCTTA	GAACTATGTGCCATCTTGGA
G6PD	CCAGCCTTCTACAAGCACCTCAA	AATAGCCCCCACAACCCTCAGTA
Gαo	GTCACCGACATCATCATTGC	AGGTTAGACAGGGGCTTGGT
Kcnj5	TCCTTCTAGTGCAGGCCATC	TTTTCCAAGGTGAGGACTGG
Kir6.1	CACAAGAACATCCGAGAGCA	CAGACTCCAGGCCACTCTTC
Nr2f2	AAAGTCCCAGTGTGCTTTGG	ATATCCCGGATGAGGGTTTC
Pitx2	GTACCCCGGCTACTCGTACA	CACCATGCTGGACGACATAC
RGS2	ATTGGAAGACCCGTTTGAGCTA	TTCCTCAGGAGAAGGCTTGATAA
RGS6	ATGTCGGCGTTTGAAGAATC	AAGCTTTCAGCCACTTTGGA
Sec22	GCAAGATGTGCAGAGGATCA	ATCTCACAGCCACCAAAACC
SGNE1	TCCAAATCCCTGTCCTCTTG	TGTCCAGCCTCTTTCCTTGT
TREK-1	GTGTCCTCTTCGTGGCTCTC	ACGAGGATCCAGAACCACAC

### Immunohistochemistry

Paraffin-embedded, paraformaldehyde-fixed rat atrial tissue sections were deparaffinized in toluene and hydrated in a graded alcohol series. The sections were incubated for 30 minutes in 3% H_2_O_2_/methanol, washed for 5 minutes in PBS, blocked with 5% normal goat serum in PBS for 60 min followed by a single wash in PBS and incubated with the primary antibody in 2% normal goat serum, 2% BSA in PBS overnight at 4°C, and then incubated for 60 min with biotinylated anti-rabbit IgG. The primary antibody dilution of secretogranin V antibody (SGNE1) (ab22699, Abcam, Cambridge, MA) was 1:500. The slides were then washed in PBS, incubated with Fluorescein Avidin D for 30 min, washed in PBS and distilled water and mounted using Vectashield mounting media (Vectorlabs), and examined with a Leica fluorescence confocal microscope.

## Authors' contributions

MFM collected the samples, performed the analysis of the microarray data, carried out the real-time RT-PCR experiments, and drafted the manuscript. AJdB participated in the design and coordination of the experiment, assisted in the interpretation, and contributed to the discussion. All authors have read and approved the final manuscript.

## Supplementary Material

Additional file 1 **Filtered signal intensities data of atrial and ventricular biological replicates**. List of 1415 differentially expressed genes. Normalized signal intensities are shown for left atrial replicates (LA (n)) and left ventricular replicates (LV (n)). The average signal intensities of each probe set within the atrial and ventricular groups were calculated and the difference between the two averages was determined (log2 ratio). Click here for file

Additional file 2 **Differentially expressed genes between normal left atrial and left ventricular tissues classified into the Ligand related functional groups**. Positive fold change values indicate a higher abundance in the atria compared to the ventricles, whereas negative fold change values demonstrate a higher ventricular abundance in comparison to the atria. Click here for file

Additional file 3 **Differentially expressed genes between normal left atrial and left ventricular tissues classified into the Receptor related functional groups**. Positive fold change values indicate a higher abundance in the atria compared to the ventricles, whereas negative fold change values demonstrate a higher ventricular abundance in comparison to the atria. Click here for file

Additional file 4 **Differentially expressed genes between normal left atrial and left ventricular tissues classified into the DNA binding protein functional group**. Positive fold change values indicate a higher abundance in the atria as compared to the ventricles, while negative fold change values demonstrate a higher ventricular abundance in comparison to the atria. Click here for file

Additional file 5 **Comparative analysis of atrial vs. ventricular differentially expressed genes from Tabibiazar et al., Kaynak et al., and Zhao et al., that are in common with Forero et al.**. Click here for file
